# Cost implications for the NHS of using the Alere™ i Influenza A & B near patient test with nasal swabs

**DOI:** 10.1186/s41512-018-0031-8

**Published:** 2018-08-01

**Authors:** A. J. Allen, R. A. O’Leary, S. Davis, S. Graziadio, W. S. Jones, A. J. Simpson, D. A. Price, L. Vale, M. Power

**Affiliations:** 1 NIHR Newcastle In Vitro Diagnostics Co-operative, Newcastle upon Tyne, UK; 2 Institute of Cellular Medicine, Newcastle University, Newcastle upon Tyne, UK; 3 Newcastle upon Tyne Hospitals NHS Foundation Trust, Newcastle upon Tyne, UK; 4 Department of Virology, Sheffield Teaching Hospitals NHS Foundation Trust, Sheffield, UK; 5 Department of Infectious Diseases, Royal Victoria Infirmary, Newcastle upon Tyne, UK; 6 Health Economics Group, Institute of Health and Society, Newcastle University, Baddiley-Clark Building, Richardson Road, Newcastle upon Tyne, UK

**Keywords:** Near-patient testing, Point-of-care testing, Influenza, Flu, Alere™ i Influenza A & B test, Cost analysis, Cost consequences, Cost minimisation, Infection control

## Abstract

**Background:**

Influenza is an acute viral infection of the respiratory tract. A rapid confirmatory diagnosis of influenza is important, since it is highly transmissible and outbreaks of influenza within the hospital setting increase morbidity and mortality. The objective of this study was to evaluate the cost implications, from the perspective of the UK NHS, of using on-label nasal swabs with the Alere™ i Influenza A & B test in a near patient setting.

**Methods:**

A cost consequence model was developed. The time horizon of the model was from hospital admission on suspicion of influenza until the end of treatment (following a diagnosis of influenza or discharge from hospital). Data on the prevalence of influenza and the sensitivity and specificity of the Alere™ i Influenza A & B test came from two prospective observational diagnostic accuracy studies. Costs were obtained from published resources. Uncertainties in the model data were investigated using deterministic, one-way sensitivity analyses.

**Results:**

Using the Alere™ i Influenza A & B point of care test with nasal swabs (on label) in NHS medical assessment units and emergency departments could save approximately £242,730 per 1000 adults presenting with influenza-like symptoms. The main cause for this was reduced times to availability of the result compared with the laboratory RT-PCR test. Other key drivers of savings were the cost of isolation, the prevalence of influenza, the specificity of the test, and the availability of isolation resources.

**Conclusions:**

The Alere™ i Influenza A & B point of care test would have greatest impact in hospitals that have extensive delays in the time to receive a result. Sensitivity analyses identified the model parameters which would have greatest effect on the result and confirmed that assumptions were conservative, i.e. did not change key results.

## Background

Influenza (flu) is a contagious viral disease that can cause mild to severe illness. Risk factors for severe complications from influenza include pregnancy, old age, and long-term conditions that impair body’s defence systems [1, 2]. Influenza is easily transmitted in healthcare environments. Key to preventing its transmission in such settings is rapid diagnosis and prompt initiation of drug treatment [3] and infection control procedures, which ideally include isolation precautions for admitted patients, as well as barrier nursing.

A recent study in the UK, conducted at three sites with on-site laboratories and one with an off-site laboratory, estimated that the median time for a swab to be taken and a result to be returned from standard respiratory virus testing was 1.2 days after a patient was admitted [4]. With delays such as this, clinicians must make initial management decisions based on clinical signs and symptoms, and then wait for the laboratory results to finalise the diagnosis and adjust management [1]. Similarities between the clinical features of influenza and other respiratory viruses make clinical diagnosis unreliable: the sensitivity of clinical judgement has been shown to be as low as 27% when compared to polymerase chain reaction (PCR)-based tests [5].

Rapid, accurate diagnostic tests for influenza, highlighted as a priority for diagnostic test development by the World Health Organization, allows influenza to be managed according to test results and avoids the empiric use of antibiotics in patients with influenza [6]. Rapid influenza antigen detection tests are popular point of care tests due to their ease of use and ability to provide a result within 15 to 30 min. However, their sensitivities tend to be low (ranging from 10 to 70%), meaning that they are not useful at excluding influenza [7]. Direct fluorescent antibody assays are more sensitive and can provide a result within 3 h, but require skilled laboratory staff. Rapid PCR-based nucleic acid amplification tests (NAATs) have been developed with equivalent sensitivity to real-time reverse transcriptase PCR (RT-PCR) and can identify influenza with a turnaround time of less than 75 min. However, they need to be performed in a laboratory [8].

The Alere™ i Influenza A & B near patient test (Alere flu NPT) uses isothermal nicking-enzyme amplification technology (NEAR) to provide a result within 15 min. A recent clinical evaluation of the Alere flu NPT with cohorts from four UK NHS adult medical assessment units (MAUs) and accident and emergency departments (A&Es) compared it with RT-PCR performed by a service laboratory. Following established practice, the study used off-label throat swabs in place of on-label nasal or nasopharyngeal swabs. The study concluded that, with the Alere flu NPT’s high specificity of 96.8% (95% confidence interval (CI) 95.2 to 98.3%) for combined influenza A and B, its use could generate substantial savings by optimising the management of patients admitted to a medical assessment unit with possible influenza. This finding provides motivation for the current study.

Further to the above, Davis et al. [4] found that the sensitivity of the Alere flu NPT was 75.8%, CI 67.0 to 84.6%. This is notably lower than that found in an American multicentre cohort study of the Alere flu NPT: sensitivity 97.8%, CI 91.6 to 99.6% for influenza A and sensitivity 91.8%, CI 82.4 to 96.6% for Influenza B, when compared with viral cell culture [9]. This study used RT-PCR to resolve discrepancies which resulted in improved diagnostic accuracy measures: sensitivity 99.8%, CI 96.8 to 100% for influenza A and sensitivity 98.1%, CI 99.3 to 97.0% for Influenza B. It was suggested that the difference in sensitivities was due to differences in sample collection: throat swabs (off-label) in the UK study and nasal swabs (on-label) in the American study. The high sensitivity with nasal swabs was confirmed in a recent prospective evaluation comparing the Alere flu NPT with another commercially available rapid isothermal NAAT [10]. The two studies providing data on test accuracy for the model were all assessed as low risk of bias [11].

Two of the centres involved in the UK clinical evaluation have since adopted the Alere flu NPT test using throat swab samples. Because the sensitivity was found to be low for this sample type, patients with a negative Alere flu NPT test are also tested for influenza with the service laboratory RT-PCR assay. This raises the question about clinical outcomes and potential cost savings if the test were to be used with nasal swabs, without supplementary service laboratory testing for negative tests.

We addressed this question by modelling the clinical outcomes and budget impact of the standard care pathway and pathways using the Alere flu NPT with throat swabs or nasal swabs.

## Methods

Suspected influenza was defined as a fever ≥ 37.8 °C and at least one influenza-like symptom: headache, extreme tiredness, dry cough, sore throat, runny or stuffy nose, and muscle pain [4].

The costs associated with the diagnosis and management of influenza were modelled for a hypothetical cohort of 1000 patients (adults and children) with suspected influenza. Costs were estimated from the perspective of the UK NHS. The cohort size was chosen so the model’s results can easily be extrapolated for actual NHS hospitals.

The total cost of a testing strategy includes the cost of isolation, the cost of antiviral prescribing, and the cost of prophylactic treatment of other patients exposed to patients being treated for influenza. Costs are presented in pounds sterling for 2017.

### Design of the cost consequence model

**Fig. 1:**
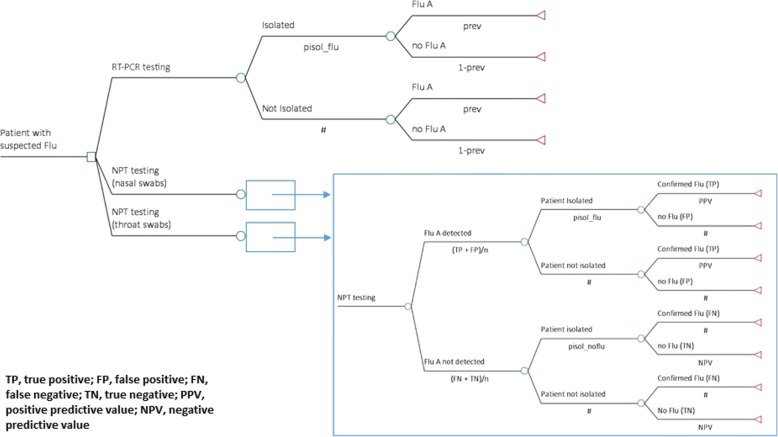
Model structure: RT-PCR testing compared with Alere flu NPT nasal and throat swab testing

A decision-analytic model, shown in Fig. 1, was developed in TreeAge Pro 2009 (© 2018 TreeAge Software Inc., Williamstown, MA, USA). For quality assurance, the analysis was also carried out using R (© 2004–2016, The R Foundation for Statistical Computing, Vienna, Austria).

The time horizon of the model is the acute episode of suspected influenza and its directly associated costs, and thus no discounting was needed.

Three strategies for diagnosing influenza were compared:

#### Strategy 1: service laboratory RT-PCR testing for influenza (current practice, and base case for comparisons)

Patients with suspected influenza in a hospital setting are investigated and treated according to the hospital’s local protocol, which will include guidance on the use of isolation measures according to availability and clinical judgement of need.

#### Strategy 2: Alere™ i Influenza A & B near patient test (new test, nasal swabs, higher sensitivity)

Following presentation with suspected influenza, the decision about treatment and isolation (subject to resource availability) is made according the result of the Alere flu NPT. A patient who is isolated based on a positive result of a near patient test will be removed from isolation when their symptoms resolve. For true positive patients, this has been estimated to be 5 days; however, for those patients with a false positive test, the duration of their symptoms is likely to be shorter, and we estimated this as 3 days.

#### Strategy 3: Alere™ i Influenza A & B near patient test (new test, throat swabs, lower sensitivity)

Following presentation with suspected influenza, the decision about treatment and isolation (subject to resource availability) is made according the result of the Alere flu NPT. People who test negative are tested with RT-PCR by the service laboratory. A patient who has tested negative for flu with the Alere flu NPT, who later receives a positive diagnosis for flu with the RT-PCR test (i.e. a false negative result for the NPT), will be isolated for the duration of the symptoms. As the full duration of isolation for a positive flu patient is 5 days and the RT-PCR result is returned within 3 days, the length of stay in isolation is estimated to be 2 days.

### Quantifying the model’s parameters

The data used for the model and their sources are shown in Table 1. PUBMED and NICE Evidence Search were used to identify health technology assessments with data relevant to the UK. When relevant data were not available in the published literature or datasets, we obtained the opinions of experts involved in the UK clinical evaluation of the Alere flu NPT [4].

**Table 1:** Model parameters and data sources

Parameter for the model	Base case value	Data source
Number of participants in study	1000	
Sensitivity of NPT with nasal swabs	0.98	Bell et al. [9]
Specificity of NPT with nasal swabs	0.86	Bell et al. [9]
Sensitivity of NPT with throat swabs	0.76	Davis et al. [4]
Specificity of NPT with throat swabs	0.97	Davis et al. [4]
Prevalence of influenza	0.17	Bell et al. [9]
Length of stay, influenza diagnosed (days)	5.00	Duration of antiviral treatment
Length of stay, influenza not diagnosed (days)	3.00	Av days to PCR result
Cost of NPT test	23.45	Estimate
Cost of RT-PCR	70.00	Estimate
Cost of full isolation (Cost of partial isolation = cIsol/2)	88.43	Health Protection Scotland [14]
Cost per dose of 75 mg oseltamivir (taken every 12 h)	1.54	MIMS [15]
Cost of nosocomial infection	2*(cOseltamivir * 2 * RxOseltamivir + cIsol*los)	NICE guidance [1]
Number of days for oseltamivir treatment	5.00	MIMS [15]
Probability of isolation if influenza detected	1.00	Expert opinion
Probability of isolation if influenza not detected	0.00	Expert opinion
Probability of nosocomial infection	0.20	Expert opinion

Diagnostic accuracy data for Strategy 2, in which nasal swabs were used for the Alere flu NPT, were obtained from a study conducted in the USA. This study compared the influenza A result from the Alere flu NPT to culture. Because no diagnostic accuracy data were available for Influenza B, the model extrapolated the sensitivity and specificity results from influenza A to influenza B.

The effectiveness of antiviral treatment is greatest if started within 48 h of the onset of symptoms [12]. Consequently, the model assumes that patients with fewer than 48 h of symptoms would receive antiviral treatment for 5 days if the RT-PCR was positive, or 3 days (the average time to RT-PCR result) if it was negative.

When published information was not available, estimates were provided by NHS clinicians, with expertise in the field of infectious disease and who were co-investigators in a recent UK clinical evaluation of the Alere flu NPT [4]. Estimates were obtained this way for the costs of antiviral treatment, the costs of isolation, the cost associated with prophylactic treatment of patients exposed to a patient with influenza (to prevent developing a nosocomial infection), and the probabilities of outcomes such as onward transmission. The cost of a transmission associated with a false negative Alere flu NPT result was estimated as the combined cost of isolation and antiviral treatment.

### Sensitivity analyses to explore uncertainties in the model’s outcomes

A series of sensitivity analyses explored the effects of uncertainties in the model’s assumptions on the model’s outcomes. One-way sensitivity analyses were carried out for each of the parameters in Table 1. The effects on patient management costs were explored in sensitivity analyses that omitted influenza testing costs. Other sensitivity analyses explored uncertainties resulting from applying the US data to NHS adult MAUs and A&Es. The proportion of people presenting more than 48 h after the onset of symptoms was not included in the sensitivity analysis (or model) since the only difference to the model’s results would be in the cost of oseltamivir which is 1.7% the cost of isolation, and therefore negligible.

Because there was no evidence or expert opinion available on the minimum and maximum values to employ in the sensitivity analysis of isolation cost (Table 1), this parameter was varied from 50 to 150%, a range that encompasses the differences in isolation costs at different facilities.

The prevalence of influenza (Table 1) was varied from that observed in the clinical evaluation (base case) to a minimum of 0 (corresponding to the start and end of the influenza season) and a maximum of 0.4 (corresponding to the peak of the seasonal epidemic in the UK). The upper limit is above the highest average single prevalence observed during the clinical evaluation of the Alere™ i Influenza A & B near patient test in four UK hospitals during the 2014/2015 influenza season [4].

The base case probability of onward transmission of nosocomial infection (Table 1) was provided by experts. The range used for the sensitivity analysis of transmission probability was from 0 (e.g. all high-risk patients exposed to influenza are vaccinated, have sufficient immunity, and therefore do not contract influenza) to 0.4 (if not all high-risk patients are vaccinated, some may contract influenza when exposed to patients with it).

The length of patient stay without confirmed influenza (Table 1) was varied for the sensitivity analysis from 1 to 5 days (i.e. 2 days around the base case assumption of 3 days). The lower limit represents the scenario in which a patient, despite suspicion of influenza, is not sufficiently unwell to be admitted to a ward and is simply sent home. The upper limit accounts for patients who do not have influenza, but return of influenza testing results is delayed.

The sensitivity and specificity of the Alere flu NPT test with both nasal (on label) and throat (off label) swabs were also varied for the sensitivity analysis by 10% in either direction.

For all parameters, the Alere flu NPT testing with both swab types results in cost savings across the ranges used in the sensitivity analyses when compared with RT-PCR testing.

## Results

Overall, Strategy 2, Alere flu NPT testing with nasal swabs (on label), is estimated to provide total savings over £242,730 for a cohort of 1000 patients with influenza-like symptoms (Table 2). Strategy 3, using the Alere flu NPT with throat swabs (off label), is less costly than current practice, with total savings of £211,857.

**Table 2:** Cost analysis of the RT-PCR and Alere™ i Influenza A & B near patient testing strategies for patients with suspected influenza. Cells with italicized text identify the NPT testing strategy that provides the lowest cost and maximum saving for that line item

	Costs per cohort of 1000 patients			
Test	RT-PCR	Alere flu NPT with:		Maximum saving
Cost of:		Nasal swab	Throat swab	
Isolation	£295,356	£104,489	*£71,298*	£231,334
Influenza testing	£70,000	*£23,450*	£82,571	£46,550
Antiviral treatment	£10,294	£4357	*£2395*	£7898
Nosocomial infection	£0	*£622*	£7529	− £622
Total cost	£375,650	*£132,919*	£163,793	£242,730

The cost components that drive these potential savings and the results of sensitivity analyses exploring the effects of the uncertainties in the model parameters are described in the following sections.

### Isolation costs

In Strategy 1, the RT-PCR testing pathway, all 1000 patients are presumptively isolated at a total cost of £295,356 before results become available. With the prevalence of influenza being 17% (an approximate average over the winter epidemic), only 170 of these patients would have flu, meaning that 830 would be isolated unnecessarily for 3 days while waiting for the RT-PCR result. The cost of this would be £220,190. With Strategies 2 and 3, Alere flu NPT testing, only those who test positive would be isolated. The total cost for the cohort is £104,489 for Strategy 2 (using nasal swabs) and £71,298 for Strategy 3 (using throat swabs), giving savings on this line item of £190,867 and £231,334 respectively. The savings are higher for Strategy 3 (using throat swabs) due to the greater number of false negative results (Strategy 2: Fn = 3, Strategy 2: Fn = 41).

### Influenza testing costs

The total costs for testing for influenza in Strategies 1 and 2 (RT-PCR and Alere flu NPT with nasal swabs) are £70,000 and £23,450 respectively. In Strategy 3 (Alere flu NPT using throat swabs), patients who test negative on the NPT are also tested for influenza using the laboratory RT-PCR at an additional cost of £59,120.

### Prescribing costs

With Strategy 1 (testing with RT-PCR), assuming that the prevalence of influenza is 17%, 830 people would receive antiviral treatment unnecessarily, at a cost of £7674 (i.e. influenza-negative patients receiving antiviral treatment until a negative test result). With Strategies 2 and 3, Alere flu NPT testing, only those who test positive receive antivirals. Of those testing positive in the nasal swab and throat swab pathways, 116 and 27 patients would have false positive results and they would receive unnecessary antiviral treatment costing £1788 and £416, respectively. Strategy 3, NPT testing with throat swabs, provides greater savings because the specificity is greater than when using throat swabs.

### Onward transmission costs

All patients in Strategy 1, RT-PCR testing, are presumptively isolated and therefore there are no costs associated with the onward transmission of the virus to other patients. For Strategies 2 and 3, Alere flu NPT testing, the nasal swab and throat swab tests are not 100% sensitive: 3 and 41 patients, respectively, would receive a false negative diagnosis. The people initially considered as negative would not be isolated and could have infected other patients. The costs associated with this onward transmission for a cohort of 1000 patients are £622 when nasal swabs are used (Strategy 2) but is substantially higher at £7529 when throat swabs are used (Strategy 3).

### Sensitivity analyses

Table 3 ranks the parameters by the range in cost savings, from highest to lowest for both swab types. Varying the average length of stay with in-patients with influenza-like symptoms, who are later confirmed to not have influenza, gives the largest change in cost savings: £262,718 for Strategy 2, Alere flu NPT with nasal swabs, and £308,977 for Strategy 3, Alere flu NPT with throat swabs. The next most influential parameters for both swab types are, in decreasing order, the cost of isolation and the probability of isolation in patients who have a positive influenza test. Varying the prevalence of influenza results in the same range of savings for both swab types. Varying the Alere flu NPT specificity has a larger effect on savings than varying the sensitivity for both swab types. However, the probability of nosocomial infection has negligible effects on the savings when nasal swabs are used and a larger effect when throat swabs are used.

**Table 3:** Univariate sensitivity analysis showing the savings (when compared to RT-PCR testing) for the minimum and maximum values of each parameter. Top panel: Strategy 2, NPT testing with nasal swabs. Bottom panel: Strategy 3, NPT testing with throat swabs

	Savings (min value of parameter)	Savings (max value of parameter)	Savings range
Strategy 2. NPT testing with nasal swabs			
Length of stay no flu (1–5)	£64,822	£327,540	£262,718
Isolation cost (£44.22–£132.65)	£100,747	£291,615	£190,867
Probability of isolation with flu (0–1)	£5936	£196,181	£190,245
Prevalence (0–0.4)	£165,067	£238,276	£73,210
Specificity nasal swabs (0.7–0.96)	£172,883	£219,479	£46,596
Sensitivity nasal swabs (0.8–1)	£200,848	£195,248	£5601
Probability of nosocomial infection (0–0.4)	£196,803	£195,559	£1245
Length of stay with flu (3–7)	£195,580	£196,782	£1203
Strategy 3. NPT testing with throat swabs			
Length of stay no flu (1–5)	£69,939	£378,916	£308,977
Isolation cost (44.215–132.645)	£112,399	£336,457	£224,059
Probability of isolation with flu (0–1)	£623	£224,428	£223,805
Prevalence (0–0.4)	£193,314	£266,523	£73,210
Specificity throat swabs (0.7–1)	£201,596	£231,883	£30,288
Probability of nosocomial infection (0–0.4)	£231,957	£216,898	£15,059
Sensitivity throat swabs (0.8–0.86)	£226,055	£222,734	£3321
Length of stay with flu (3–7)	£224,428	£224,428	£0

Due to the low sensitivity of the Alere flu NPT when throat swabs are used, a clinician may choose not to remove a patient from isolation until a negative RT-PCR result is obtained. In this scenario, the cost of isolation for Strategy 3, Alere flu NPT with throat swab, would be the same as that for Strategy 1, RT-PCR testing (£295,365 for cohort of 1000 patients), and would be the most costly strategy overall (£380,321 for a cohort of 1000 patients).

Our analysis assumes that all patients suspected of influenza would be isolated. However, in practice, isolation resources are limited and their availability can change over the course of an epidemic, e.g. when wards are temporarily allocated for the sole use of patients with influenza (“cohorting”). Sensitivity analysis found that isolation rates between 0 and 1 had a substantial effect on the cost savings made possible with the use of the Alere™ i Influenza A & B near patient test. When the probability of isolation was low, e.g. when isolation resources are extremely limited, savings from using the Alere flu NPT with both swab types were predicted to be modest (£5936 for nasal swabs and £623 for throat swabs).

## Discussion

When a patient is admitted to A&E or MAU for suspect influenza, the standard practice in the UK NHS is to confirm the diagnosis with RT-PCR test for influenza. This testing strategy was compared in a decision model with approaches that employ the Alere™ i Influenza A & B near patient test used with either throat swabs (off-label, but common practice in the UK NHS) or nasal swabs (on-label, and usual practice in the USA). The reason given by NHS clinicians for preferring throat swabs is that nasal swabs are more uncomfortable for patients and more difficult for clinicians. Our modelling methods followed (with some minor modifications) the guidelines published by the International Society For Pharmacoeconomics and Outcomes Research [13]. Sensitivity analyses were designed to include the range of parameter values relevant to decision makers in the UK NHS.

The Alere flu NPT was found to save costs compared to service laboratory RT-PCR testing, regardless of swab type used. However, the cost savings are slightly greater when nasal swabs (on label) are used. This is primarily due to the higher sensitivity with nasal swabs, as found in previous clinical evaluations [9, 10].

The greatest impact on costs comes from reducing the time to availability of the result and the subsequent increased accuracy in employing isolation measures and prescribing antivirals.

The lower sensitivity of the Alere flu NPT used with (off label) throat swabs resulted in the model predicting a larger number of patients with false negative test results, who would not be treated or isolated until the laboratory results became available, with the result that influenza would be transmitted to other patients.

The model assumes that isolation precautions are not used for patients who test negative for flu. Although isolation measures would be indicated for some of these patients, in the A&E and MAU this is a rare occurrence and would have negligible effects on the model’s outcomes.

The assumptions made in the model are conservative, i.e. making its structure more sophisticated would be unlikely to change conclusions drawn from its results. For example, the operational definition of “possible influenza” had a low threshold for including people with increased respiratory symptoms and chronic heart failure or chronic obstructive pulmonary disease.

### Limitations

The data in our analysis are from observational studies. The results therefore indicate what could be achieved if tests are used and results applied as modelled. A randomised controlled interventional study would be able to assess if the model’s assumptions translate into reality. (Given the cost of the research and the growing adoption of rapid point tests, such research is unlikely to happen.)

Our analysis considers facility-specific costs over a relatively short time horizon. This choice was made in order to assess the costs directly associated with the admission of a patient with suspected influenza, and does not take into account costs or benefits associated with other possible reasons for admission. Costs of complications (e.g. costs of extended inpatient stay or higher levels of care) were not included. The costs are high, but infrequently incurred. Omitting the costs of complications from the model is conservative, in that their exclusion biases results in favour of the less sensitive test method, throat swabbing, since differences in costs would come from differences in onward transmission from patients with flu who were not isolated.

The main structural limitations in the model are the simplification of the effects of transmitting influenza, not including other testing strategies for comparison, and not taking account of patients with an influenza-like illness who would be isolated even if they test negative for flu. However, it would have been outside the aims and resources of this project to extend its scope, for example to the public health goal of limiting the spread of infection.

A probability sensitivity analysis (PSA) was not conducted as it was outside the available resources for this project. The main drivers of savings identified in the one-way exploratory sensitivity analysis were reduced times to availability of the result, cost of isolation, prevalence of influenza, specificity of the test, and the availability of isolation resources, and it seems unlikely that a PSA would have changed the study’s conclusions.

## Conclusions

The higher sensitivity when using the Alere™ i Influenza A & B test with (on label) nasal swabs has the potential to increase savings for MAUs and A&Es in the UK NHS and, importantly, reduce onward transmission of influenza. Future research should repeat the diagnostic performance evaluation for the Alere™ i Influenza A & B near patient test using both nasal and throat swabs in the same cohort. This would, of course, involve addressing the issue of obtaining informed consent and ethical approval for multiple swabbing of the same patient.

Future research should also evaluate outcomes from the short time to result with a rapid influenza such as the potential for earlier antiviral use, fewer antibiotics for patients with confirmed influenza, reduction of unnecessary investigations, more appropriate discharge of patients, and potential harms.

## Abbreviations

A&E: Accident and emergency department
CI: Confidence interval
FN: False negative
FP: False positive
IVD: In vitro diagnostic
MAU: Medical assessment unit
NAAT: Nucleic acid amplification tests
NEAR: Nicking-enzyme amplification technology
NPT: Near patient test
NPV: Negative predictive value
PCR: Polymerase chain reaction
PPV: Positive predictive value
RT-PCR: Real-time reverse transcriptase polymerase chain reaction
TN: True negative
TP: True positive

## References

[1] NICE Clinical Knowledge summaries. Influenza - seasonal http://cks.nice.org.uk/influenza-seasonal. Accessed 22 May 2018.

[2] Mertz D, et al. Populations at risk for severe or complicated influenza illness: systematic review and metaanalysis. Br Med J. 2013;347:f5061. 10.1136/bmj.f5061 PMC3805492 23974637

[3] Jefferson T, et al. Oseltamivir for influenza in adults and children: systematic review of clinical study reports and summary of regulatory comments. Br Med J. 2014;348:g2545. 10.1136/bmj.g2545 PMC3981975 24811411

[4] Davis S (2017). Diagnostic accuracy and cost analysis of the Alere™ i Influenza A&B near-patient test using throat swabs. J Hosp Infect.

[5] Dugas AF (2015). Clinical diagnosis of influenza in the ED. Amer J of Em Med.

[6] Brendish N, Schiff H, Clark T (2015). Point-of-care testing for respiratory viruses in adults: the current landscape and future potential. J Infect.

[7] Hannoun C, Tumova B, European Scientific Working group on Influenza (2000). Survey on influenza laboratory diagnostic and surveillance methods in Europe. Eur J Epidemiol.

[8] Salez N (2015). Prospective and retrospective evaluation of the Cepheid Xpert® flu/RSV XC assay for rapid detection of influenza A, influenza B, and respiratory syncytial virus. Diagn Microbiol Infect Dis.

[9] Bell J (2014). Multicenter clinical evaluation of the novel Alere™ i Influenza A&B isothermal nucleic acid amplification test. J Clin Virol.

[10] Nguyen Van JC, et al. Prospective evaluation of the Alere i Influenza A&B nucleic acid amplification versus Xpert Flu/RSV. Diagn Microbiol Infect Dis. 2016;85(1):19–22. 10.1016/j.diagmicrobio.2015.11.012 26899154

[11] Whiting PF (2011). QUADAS-2: a revised tool for the quality assessment of diagnostic accuracy studies. Ann Intern Med.

[12] Amantadine, oseltamivir and zanamivir for the treatment of influenza Technology appraisal guidance. 2009. Available from: https://www.nice.org.uk/guidance/ta168. Accessed 22 May 2018.

[13] Sullivan SD (2014). Budget impact analysis—principles of good practice: report of the ISPOR 2012 budget impact analysis good practice II task force. Value Health.

[14] Health Protection Scotland. NHS Scotland MRSA Screening Pathfinder Programme Final Report Volume 2: An assessment of the economics, implementation and modelling of universal MRSA screening. 2011. http://www.documents.hps.scot.nhs.uk/hai/mrsa-screening/pathfinder-programme/mrsa-pathfinder-vol2-2011-02-23.pdf. Accessed 23 May 2018.

[15] Monthly Index of Medical Specialties (MIMS). 2014 Available at: www.mims.co.uk/drugs/infections-and-infestations/viral-infections/tamiflu. Accessed 12 June 2018.

